# Development and validation of nomograms for predicting axillary non-SLN metastases in breast cancer patients: A retrospective analysis

**DOI:** 10.3389/fonc.2023.1096589

**Published:** 2023-03-10

**Authors:** Huizi Lei, Pei Yuan, Changyuan Guo, Jianming Ying

**Affiliations:** National Cancer Center/National Clinical Research Center for Cancer/Cancer Hospital, Chinese Academy of Medical Sciences and Peking Union Medical College, Beijing, China

**Keywords:** breast cancer, sentinel lymph nodes, MSKCC, nomogram, metastasis

## Abstract

**Purpose:**

The aim of this study was to develop a nomogram for predicting positive non-sentinel lymph nodes (non-SLNs) in positive SLN breast cancer patients and validate the Memorial Sloan-Kettering Cancer Center (MSKCC) nomogram for non-SLN metastasis in Chinese patients.

**Methods:**

The pathological features of 2,561 breast cancer patients were retrospectively reviewed, and the patients were divided into training and validation cohorts. Positive non-SLN predictors were identified using univariate and multivariate analyses and used to construct the nomogram. In patients with positive SLNs, the MSKCC nomogram was used to calculate the probability of non-SLN metastasis. The area under the receiver operating characteristic curve (AUC) was calculated to assess the accuracy of this model and the MSKCC nomogram.

**Results:**

According to multivariate logistic regression analysis, the number of positive and negative SLNs, tumor stage, lymphovascular invasion, perineural invasion, and extracapsular extension were independent predictive factors for non-SLN metastasis and were selected to establish the nomogram for predicting positive non-SLNs. This nomogram performed favorably in predicting positive non-SLNs, with AUCs of 0.765 and 0.741 for the training and validation cohorts, respectively. The MSKCC nomogram predicted non-SLN metastasis with an AUC of 0.755.

**Conclusion:**

A nomogram was developed and validated to assist clinicians in evaluating the likelihood of positive non-SLN. For Chinese patients with a known ER status before surgery, the MSKCC nomogram can be used to predict non-SLN metastases.

## Introduction

Axillary lymph node metastasis is an important prognostic factor in breast cancer patients. Since its introduction in the 1990s, sentinel lymph node biopsy (SLNB) has revolutionized surgeries for predicting ALN status, especially for those with clinically negative nodes. Axillary lymph node dissection (ALND) is no longer necessary when there is no metastasis in the SLNs, and thus, its surgical-associated complications can be avoided. In contrast, patients with positive SLNs require ALND. The Z0011 designed by the American College of Surgeons Oncology Group (ACOSOG) demonstrated that ALND does not prolong survival in patients with T1 to T2 breast cancer who have ≤2 positive SLNs. However, ALND is highly recommended when metastatic disease is found in more than two SLNs or when metastatic lymph nodes are identified intraoperatively (1, 2). The Z0011 trial suggested that some positive SLN patients failed to experience benefits. This result was also confirmed in China; a prospective single-arm study showed that ALND could be avoided for patients eligible for Z0011 in China (3). Therefore, unnecessary ALND may be minimized by analyzing the factors influencing non-SLN status among patients with positive SLNs.

In recent years, several prediction models have been developed using a combination of statistically significant factors, such as the Memorial Sloan-Kettering Cancer Center (MSKCC) nomogram (4), the Tenon scoring system (5), the Louisville scoring system (6), and the Stanford nomogram (7). The MSKCC nomogram is most commonly used to predict non-SLN status. However, the application range of the MSKCC nomogram is restricted because it has not yet been widely validated in Chinese populations, and the ER status of most Chinese patients is unknown at the time of surgery because diagnostic methods are different from those in other countries.

In the present study, we aimed to use a large number of patients to assess the predictive accuracy of the MSKCC nomogram and to establish a separate nomogram to identify the predictors of non-SLN status in patients with positive SLNs and use it to subsequently predict which patient subgroups might avoid ALND.

## Materials and methods

### Case selection

A total of 2,561 patients diagnosed with breast cancer between 2011 and 2022 were selected from Cancer Hospital, Chinese Academy of Medical Sciences (CAMS). The inclusion criteria were as follows: (i) diagnosis of invasive ductal carcinoma and invasive lobular carcinoma; (ii) previous lumpectomy or mastectomy; (iii) positive SLNs (macrometastases) and previous ALND; and (iv) confirmed T1–T2 stage cancer. Patients who had undergone primary systemic therapy were excluded.

**Figure 1:**
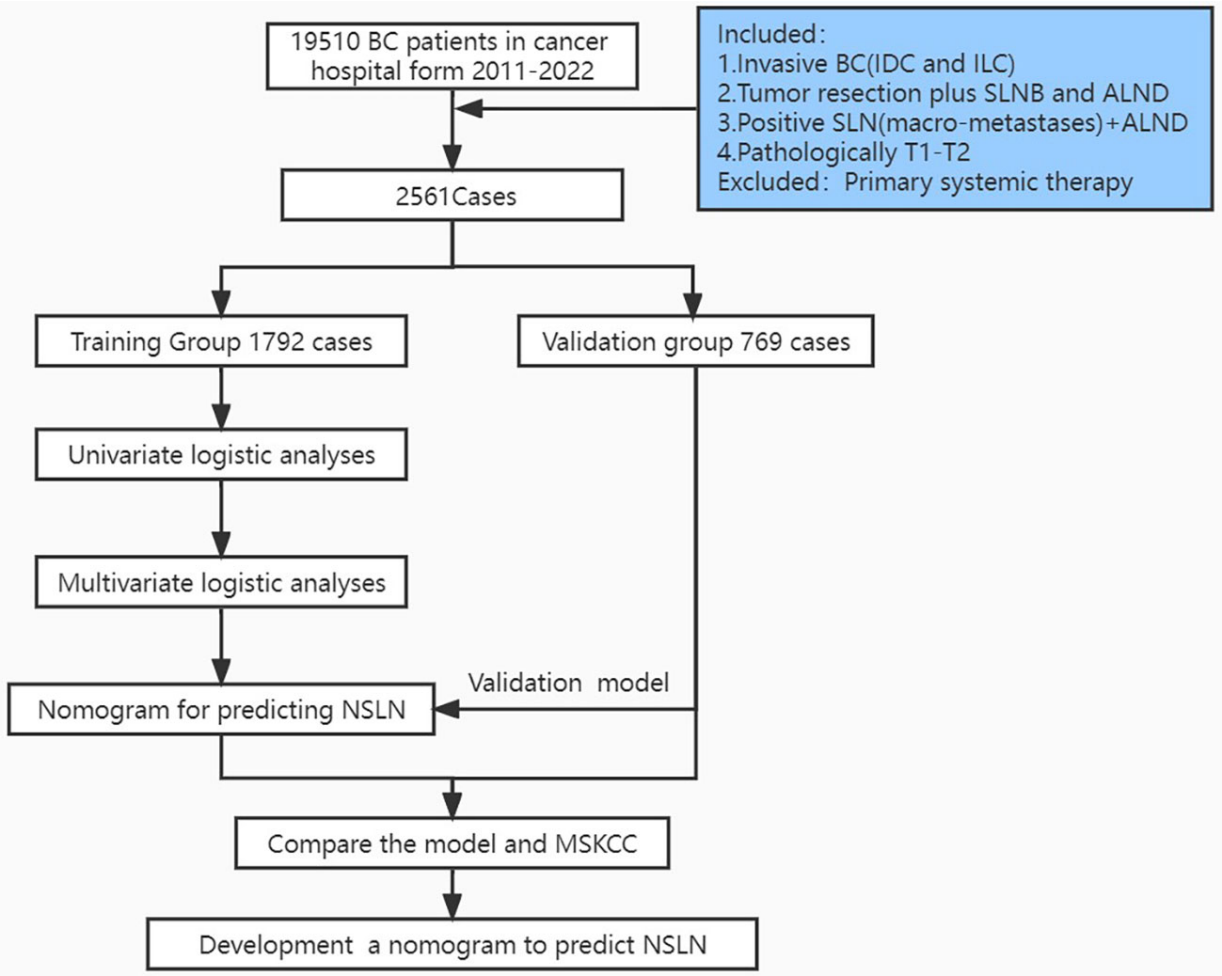
Flowchart illustrating the establishment and validation of nomograms for predicting non-SLN metastases in patients with SLN metastases.

The patients were divided into two cohorts, the training cohort (70%, 1,792/2,561) and the validation cohort (30%, 768/2,561), with the R function “createDataPartition” to ensure that outcome events were distributed randomly between the two cohorts. The prognostic risk model was constructed based on the training cohort and confirmed in the validation cohort. Thirteen variables were included: number of positive and negative SLNs, age (at diagnosis), pathological patterns, tumor stage, molecular subtype, lymphovascular invasion, perineural invasion, extracapsular extension, number of tumors, human epidermal growth factor receptor (HER2), estrogen receptor (ER), and progesterone receptor (PR). The flowchart illustrating the establishment and validation of the nomograms for predicting non-SLN metastases in patients with SLN metastases is shown in **Figure 1**.

### SLN biopsy

SLNs can be identified with nanocarbon dyes or technetium-99 m colloids. All lymph nodes detected based on radioactivity or that were dyed black were excised as SLNs for histopathological evaluation. Eight-micrometer-thick frozen sections of tumor tissue were prepared. The remaining tissue was fixed in 10% neutral buffered formalin and embedded in paraffin. Hematoxylin and eosin (H&E) staining was performed on frozen sections or on 4-μm-thick paraffin sections.

### Testing the Memorial Sloan-Kettering Cancer Center nomogram

To assess the performance of the MSKCC nomogram in predicting non-SLN metastasis, we applied it to all patients in this study. We input eight variables to the website (https://nomograms.mskcc.org/breast/BreastAdditionalNonSLNMetastasesPage.aspx) to produce an estimate of the risk of non-SLN metastasis: method of SLN metastasis detection [frozen section, routine or serial H&E, or immunohistochemistry (IHC)], pathological tumor size, tumor type and grade (ductal grade I or ductal grade II or ductal grade III or lobular), number of positive SLNs, number of negative SLNs, lymphatic or vascular structure involvement (positive or negative), multifocality (positive or negative), and ER status (positive or negative).

### Statistical analysis

Univariate analysis was performed with the Pearson chi-square test for categorical variables and independent samples *t*-tests for quantitative data. Variables with a *p-*value < 0.05 in the univariate analysis were included in binary multivariable logistic regression analysis, and multicollinearity between variables was assessed to build the clinical factor model. The potential for multicollinearity was tested using the variance inflation factor (VIF); variables with a VIF >10 were excluded from the model. Receiver operating characteristic (ROC) curves and area under the curve (AUC) values were computed using the “pROC” R package. The predicted and actual observed outcomes of the nomogram were plotted to create a calibration curve, where the 45° line represents the best prediction. The proposed nomogram was validated in an independent external validation cohort. Variables or differences with two-tailed *p-*values < 0.05 were considered statistically significant. Statistical analysis was performed using SPSS version 23.0 (IBM SPSS Statistics for Windows) and R programming language and environment (https://www.r-project.org).

## Results

### Clinical factors of the patients

The clinical characteristics of the patients are summarized in **Table 1**. The median ages were similar in the training and validation groups (50.63 ± 10.34 vs. 51.11 ± 10.66). A total of 12,434 SLNs were detected in 2,561 patients, with an average of 4.86 ± 2.00 SLNs per patient; of these, 4,616 sentinel nodes were positive, with an average of 1.80 ± 1.22 per patient. A total of 1,586 patients (61.9%) had positive axillary lymph nodes after completion of ALND, and 975 patients (38.1%) had negative lymph nodes.

**Table 1:** Clinical and pathological characteristics of the training cohort and the validation cohort.

Characteristics	Training cohort	Validation cohort
Cases	1,792	768
Positive SLN number	1.84 ± 1.28	1.71 ± 1.08
Negative SLN number	3.08 ± 2.06	2.98 ± 1.97
Age		
<50	870 (48.5)	361 (47.0)
≥50	922 (51.5)	407 (53.0)
IDC histological grade		
I	147 (8.4)	52 (7.1)
II	1,172 (67.1)	479 (65.0)
III	418 (23.9)	204 (27.7)
Unknown	10 (0.6)	2 (0.3)
Pathological patterns		
IDC	1,747 (97.5)	737 (96.0)
Invasive lobular carcinoma	45 (2.5)	31 (4.0)
pT stage		
T1	1,010 (56.4)	423 (55.1)
T2	782 (43.6)	345 (44.9)
Molecular subtype		
Luminal A	718 (40.1)	290 (37.8)
Luminal B	705 (39.3)	309 (40.2)
HER2	120 (6.7)	46 (6.0)
TNBC	104 (5.8)	49 (6.4)
Unknown	145 (8.1)	74 (9.6)
Lymphovascular invasion		
Negative	582 (32.5)	254 (33.1)
Positive	796 (44.4)	330 (43.0)
Unknown	414 (23.1)	184 (24.0)
Perineural invasion		
Negative	929 (51.8)	399 (52.0)
Positive	457 (25.5)	186 (24.2)
Unknown	406 (22.7)	183 (23.8)
Extracapsular extension		
Negative	1,692 (94.4)	733 (95.4)
Positive	100 (5.6)	35 (4.6)
Mutifocal		
No	1,574 (87.8)	683 (88.9)
Yes	218 (12.2)	85 (11.1)
HER2 IHC		
0	380 (21.2)	184 (24.0)
1+	492 (27.5)	180 (23.4)
2+	557 (31.1)	246 (32.0)
3+	279 (15.6)	119 (15.5)
Unknown	84 (4.7)	39 (5.1)
HER2		
IHC 0,1+,2+(FISH-)	1,256 (70.1)	533 (69.4)
ICH 3+,2+(FISH+)	351 (19.6)	141 (18.4)
IHC 2+(FISH Unknown) and HER2 Unknown	185 (10.3)	94 (12.2)
ER		
Negative	259 (14.5)	129 (16.8)
Positive	1,451 (81.0)	600 (78.1)
Unknown	82 (4.6)	39 (5.1)
PR		
Negative	306 (17.1)	172 (22.4)
Positive	1,402 (78.2)	557 (72.5)
Unknown	84 (4.7)	39 (5.1)

IDC, invasive ductal carcinoma; TNBC, triple-negative breast cancer.All values are *n* (%).

### Clinicopathological feature selection and nomogram building

**Figure 2:**
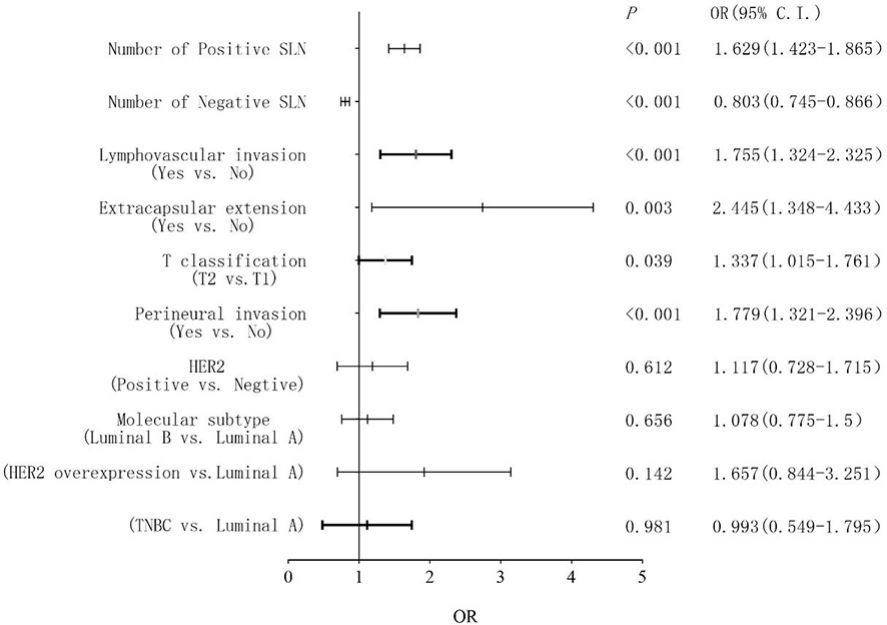
Forest plots showing the results of the multivariate logistic analysis.

Univariate analysis demonstrated that non-SLN metastasis was significantly correlated with the number of positive and negative SLNs, tumor size, tumor stage, molecular subtype, lymphovascular invasion, perineural invasion, extracapsular extension, and HER2 status (**Table 2**). The VIF values were all <10, indicating that no collinearity existed between the predictor variables. In multivariate logistic regression analysis, the number of positive and negative SLNs (*p* < 0.001), tumor stage (*p* = 0.039), lymphovascular invasion (*p* < 0.001), perineural invasion (*p* < 0.001), and extracapsular extension (*p* = 0.003) were identified as independent predictive factors for non-SLN metastasis (**Figure 2**). These six independently predictive factors were used to create a predictive nomogram.

**Table 2:** Univariate analyses of positive non-sentinel lymph nodes in the training group.

Characteristics	Training cohort (*N* = 1792)		Univariable
	Negative NSLN	Positive NSLN	*p*
Tumor size	2.03 ± 0.99	2.26 ± 0.63	<0.001
Positive SLN number	1.48 ± 0.82	2.41 ± 1.60	<0.001
Negative SLN Number	3.53 ± 2.0	2.38 ± 1.95	<0.001
Age			0.911
<50	529 (48.4)	341 (48.7)	
≥50	563 (51.6)	359 (51.3)	
IDC histological grade			0.118
I	99 (9.3)	48 (7.1)	
II	720 (67.9)	452 (66.9)	
III	242 (22.8)	176 (26.0)	
Pathological patterns			0.896
IDC	1065 (97.5)	682 (97.4)	
Invasive lobular carcinoma	27 (2.5)	18 (2.6)	
pT stage			<0.001
T1	670 (61.4)	340 (48.6)	
T2	422 (38.6)	360 (51.4)	
Molecular subtype			0.005
Luminal A	471 (46.6)	247 (43.6)	
Luminal B	403 (39.9)	302 (47.5)	
HER2	68 (6.7)	52 (8.2)	
TNBC	69 (6.8)	35 (5.5)	
Lymphovascular invasion			<0.001
Negative	422 (51.0)	160 (29)	
Positive	405 (49.0)	391 (71)	
Perineural invasion			<0.001
Negative	622 (72.1)	307 (58.7)	
Positive	241 (27.9)	216 (41.3)	
Extracapsular extension			<0.001
Negative	1055 (96.6)	637 (91.0)	
Positive	37 (3.4)	63 (9.0)	
Mutifocal			0.387
No	965 (88.4)	609 (87.0)	
Yes	127 (11.6)	91 (13.0)	
HER2 IHC			0.028
0	234 (22.3)	146 (22.2)	
1+	328 (31.2)	164 (24.9)	
2+	327 (31.1)	230 (35.0)	
3+	161 (15.3)	118 (17.9)	
HER2			0.037
IHC 0,1+,2+(FISH-)	789 (79.9)	467 (75.4)	
ICH 3+,2+(FISH+)	199 (20.1)	152 (24.6)	
ER			
Negative	158 (15.0)	101 (15.3)	0.886
Positive	892 (85.0)	559 (84.7)	
PR			
Negative	183 (17.4)	123 (18.7)	0.507
Positive	867 (82.6)	535 (81.3)	

### Internal performance and independent validation of the nomogram

**Figure 3:**
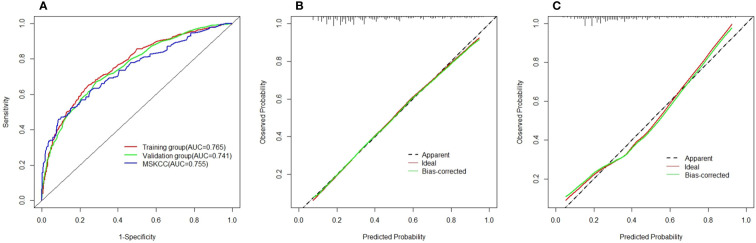
**(A)** Area under the receiver–operator characteristic curve for the training group (red) and validation group (green) and the Memorial Sloan-Kettering Cancer Center (MSKCC) nomogram (blue). **(B)** Calibration curves for the nomogram in the training group. **(C)** Calibration curves for the nomogram in the validation group.

**Figure 4:**
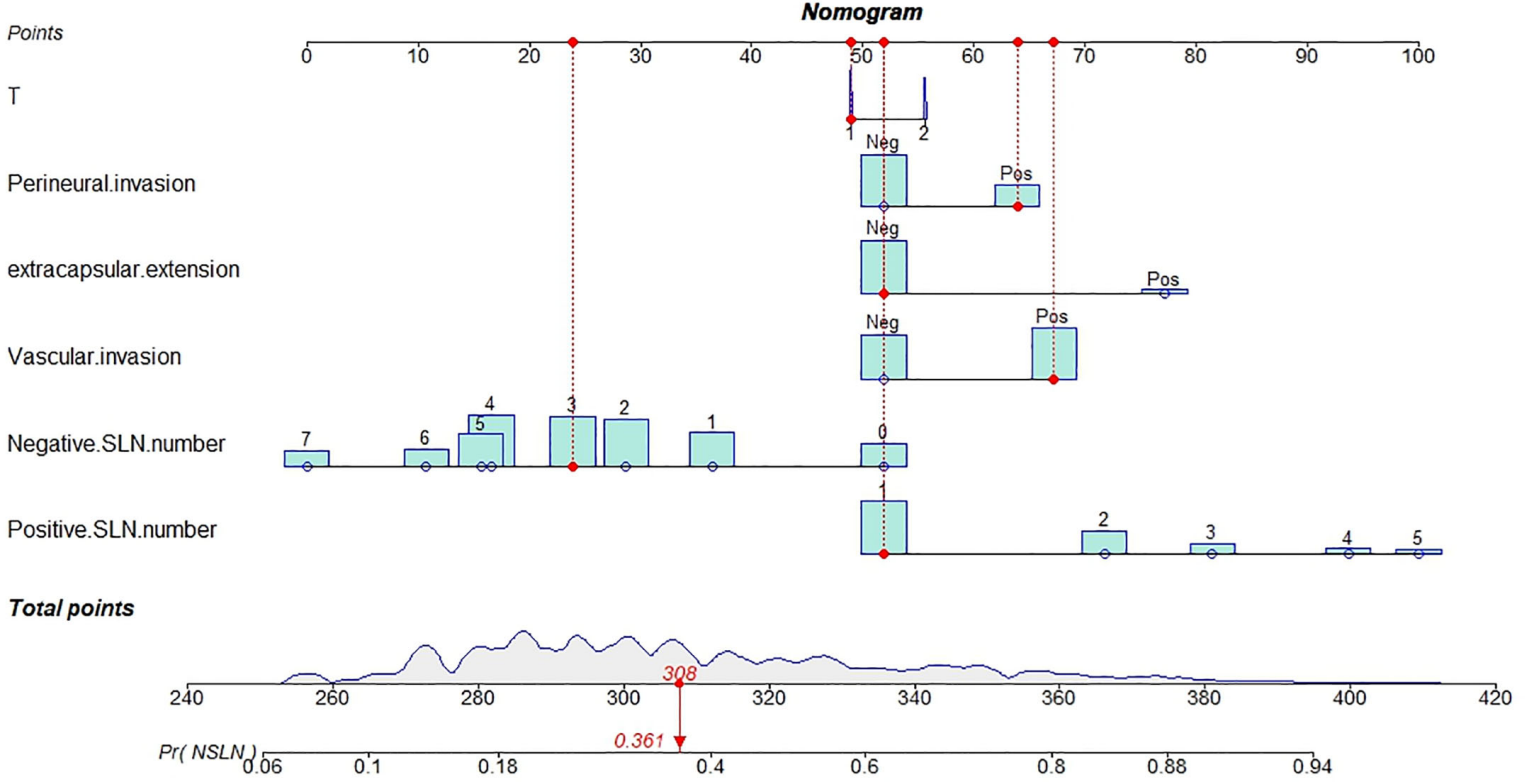
Constructed nomogram for predicting the risk of non-SLN metastasis in a patient.

The outstanding discriminability of the nomogram gave an AUC of 0.765 (95% CI: 0.738–0.793) in the training group and 0.741 (95% CI: 0.695–0.787) in the validation group (**Figure 3A**). In addition, the calibration curve of the nomogram showed good agreement between the predicted and actual observations in the training group (**Figure 3B**, *p* = 0.960) and validation group (**Figure 3C**, *p* = 0.993). In conclusion, the predictive model had good discriminative and calibration abilities. **Figure 4** shows an example of using the nomogram to predict the risk of non-SLN metastasis in a given patient. The total score was derived from the individual scores calculated using the nomogram; most patients in the training group had total risk points ranging from 260 to 380.

This patient had T1 stage breast cancer, perineural and vascular invasion, one positive surgical lymph node, and three negative surgical lymph nodes but no extracapsular extension. The density plot of total points and tumor stages shows their distribution. For category variables, their distributions are reflected by the size of the box (for perineural invasion, the smaller box represents positive, and the larger one represents negative). The importance of each variable is ranked according to the standard deviation along the nomogram scales. An individual patient’s score (black dot) is placed on each variable axis. Red lines and dots are drawn upward to determine the points received by each variable; the sum (308) of these points is located on the total points axis, and a line is drawn downward to the NSLN axes to predict the risk of non-SLN metastasis, which for this patient is 36.1%.

### Performance of the MSKCC nomogram in our cohort of SLN-positive patients

The MSKCC nomogram was used to estimate non-SLN metastasis risk in all patient groups (training and validation), with an AUC of 0.755 (95% CI: 0.732–0.778) (**Figure 3A**).

## Discussion

This study used data from 2,561 early breast cancer patients in two cohorts and presented a simple nomogram that demonstrated strong discriminability for axillary non-SLN metastases. The current trends in surgery for breast cancer are toward more conservative management, which aims to avoid the complications of ALND, such as lymphedema of the arm and restriction of arm mobility. If metastasis is not found in the SLN, further ALND is generally not needed; otherwise, the standard management is completion of the ALND. This study found that 61.66% (1,579/2,611) of patients with positive SLNs had no further non-SLN metastases. By extension, the percentage of negative non-SLNs in patients with one or two SLN metastases was 74.64% (1,077/1,443) and 57% (350/614), respectively, while in patients with three or more SLN metastases, 30.16% (152/504) did not have non-SLN metastasis. Therefore, completing ALND would have no therapeutic value in more than half of patients with SLN metastasis; this would require identifying the non-SLN low-risk subgroup to avoid unnecessary treatment. Univariate and multivariate analyses were used to assess the association between the clinical pathologic variables and non-SLN metastasis. The results showed that the number of positive and negative SLNs, tumor stage, lymphovascular invasion, perineural invasion, and extracapsular extension were independent risk factors for non-SLN metastasis. While some research has shown that non-SLN metastasis is associated with the number of tumor lesions in breast cancer, no association was observed in our study (8). In the MSKCC nomogram, the effect of ER status was only borderline significant (*p* = 0.08), but ER status was included in the MSKCC nomogram to improve the overall predictive capacity (4). Therefore, only the patients with known ER status can use the MSKCC nomogram. However, our study did not observe significant associations between ER status and NSLN metastasis (*p* = 0.886), similar to the result shown by other studies (8–10). Thus, we did not include ER status to establish the nomogram for predicting positive non-SLNs. The results of this study can not only help guide clinicians in predicting the risk of axillary non-SLN metastases and selecting appropriate treatment strategies but also provide a basis for guiding clinical decision-making in the radiation field.

SLN biopsy requires the collaboration of a multidisciplinary team of doctors to integrate and interpret clinical information. Chemotherapy and radiotherapy can be used instead of ALND in T1–T2 stage patients who have not undergone neoadjuvant chemotherapy, are clinically node negative, and have fewer than or equal to two positive SLNs (11). The number of studies on breast cancer patients with three or more positive SLNs is limited at present. Whether patients with three or more positive SLNs could receive ALND still requires confirmation with a large prospective, randomized controlled trial. Two studies randomizing patients with micrometastatic SLN to complete ALND or clinical follow-up included patients who had undergone mastectomy. Neither study showed significant effects on survival between groups, suggesting that ALND and radiotherapy are unnecessary for these patients (12, 13). ALND is also not recommended for patients with isolated tumor cells in lymph nodes (14, 15).

The MSKCC nomogram is the most widely used nomogram to predict the likelihood of non-SLN disease, using nine identified risk factors to achieve AUCs of 0.76 (retrospective group) and 0.77 (prospective group) (4). There is a great deal of variation in its predictive value among different populations. The MSKCC nomogram has been tested in many studies; some reported that the MSKCC nomogram had an AUC ranging from 0.73 to 0.80 (16, 17), but research from China reported values ranging from 0.677 to 0.688 (18–20). Likely due to the smaller validated sample size, these Chinese studies showed predictive abilities that were lower than those of the original research study. In this study, the MSKCC nomogram was applied to 1,760 patients with a positive SLN who subsequently completed ALND. The AUC value was 0.755, which is basically consistent with the original study. Although there are differences in race, age of onset, and staining methods, the prediction of metastasis in non-SLNs is also feasible with the MSKCC nomogram. A limitation of this approach is that limited pathologic information is available at the time of mastectomy. We observed a similar AUC value between our research and the initial MSKCC nomogram study, but the MSKCC nomogram cannot be widely applied to Chinese patients since the patient’s ER status is often unknown before surgery.

Of course, there were still several limitations to our study. First, only routine pathological examination and H&E-stained SLNs and non-SLNs were examined. Multisection analysis and IHC in lymph node staging may help increase the accuracy of lymph node analysis. Second, patients with lymph node micrometastases were not included in the study. Furthermore, the size of the metastatic foci in the node was unknown.

In conclusion, the nomogram we proposed uses six variables: the number of positive and negative SLNs, tumor stage, lymphovascular invasion, perineural invasion, and extracapsular extension. This nomogram can be used to estimate the likelihood of having at least one positive non-SLN in patients with positive SLNs during the surgery. An evaluation of the model showed good predictiveness, suggesting that it can be used by the surgeon in determining which surgical modality will be used. The MSKCC nomogram can be applied to Chinese breast cancer patients with known ER status before surgery, and its predictive ability was similar to that of a previous study predicting non-SLN metastases.

## Data availability statement

The original contributions presented in the study are included in the article/supplementary material. Further inquiries can be directed to the corresponding author/s.

## Ethics statement

This study involving human participants was reviewed and approved by the NCC Ethics Committee/Institution Review Board. Written informed consent for participation was not required for this study in accordance with the national legislation and the institutional requirements.

## Author contributions

HL: data curation, formal analysis, writing—original draft, and methodology. PY: data curation, writing—original draft, and formal analysis. CG: data curation, writing—original draft, and formal analysis. JY: conceptualization, resources, formal analysis, supervision, funding acquisition, investigation, methodology, writing—original draft, project administration, and writing—review and editing. All authors contributed to the article and approved the submitted version.
